# A Cell-Based Approach to Dental Pulp Regeneration Using Mesenchymal Stem Cells: A Scoping Review

**DOI:** 10.3390/ijms22094357

**Published:** 2021-04-22

**Authors:** Sahng G. Kim

**Affiliations:** Division of Endodontics, Columbia University College of Dental Medicine, New York, NY 10032, USA; sgk2114@cumc.columbia.edu

**Keywords:** mesenchymal stem cells, pulp regeneration, stem cells, cell transplantation, regenerative endodontics

## Abstract

Despite the recent explosion of investigations on dental pulp regeneration using various tissue engineering strategies, the translation of the findings from such studies into therapeutic applications has not been properly achieved. The purpose of this scoping review was to systematically review the efficacy of mesenchymal stem cell transplantation for dental pulp regeneration. A literature search was conducted using five electronic databases from their inception to January 2021 and supplemented by hand searches. A total of 17 studies, including two clinical trials and 15 animal studies using orthotopic pulp regeneration models, were included for the review. The risk of bias for the individual studies was assessed. This scoping review demonstrated that the regeneration of vascularized pulp-like tissue was achieved using the stem cell transplantation strategy in animal models. Autologous cell transplantation in two clinical studies also successfully regenerated vascularized vital tissue. Dental pulp stem cell subpopulations, such as mobilized dental pulp stem cells, injectable scaffolds such as atelocollagen, and a granulocyte-colony forming factor, were the most commonly used for pulp regeneration. The overall risk of bias was unclear for animal studies and was moderate or judged to raise some concerns for clinical studies. More high-quality clinical studies are needed to further determine the safety and efficacy of the stem cell transplantation strategy for dental pulp regeneration.

## 1. Introduction

Dental pulp regeneration requires an integrated use of three key elements, including cells, biomaterial scaffolds, and signaling molecules, which enables the recapitulation of biological processes for normal tissue development [1,2,3,4,5,6]. Various combinations of this triad have been used to regenerate the pulp–dentin complex [3,4,5]. Among them, transplantation of mesenchymal stem cells with the aid of biomaterial scaffolds or signaling molecules has been used as a major tissue engineering strategy [5]. This approach is based on the beneficial effects of transplanted stem cells, which can augment the regenerative processes of a variety of tissues [7,8,9,10,11]. The transplanted stem cells regenerate the parenchyma of a tissue as a building block following cell differentiation [12,13]. Furthermore, the stem cells release a multitude of biological trophic factors [14], which can modulate the immune function and promote regenerative cellular events, such as mobilization, proliferation, and differentiation of resident cells, in addition to enhancing angiogenesis and neurogenesis [15,16]. 

Due to stem cell plasticity induced by cell reprogramming in response to local instructive cues [17,18], various types of stem cells can be candidates for dental pulp regeneration. However, there still remains a degree of lineage commitment and differentiation associated with adult mesenchymal stem cells [18]. Tissue-specific mesenchymal stem cells such as dental pulp stem cells (DPSC) and their subpopulations have been most widely used to regenerate the pulp–dentin complex [19,20,21,22,23,24,25,26,27,28,29,30,31,32]. Other mesenchymal stem cells such as bone marrow stem cells (BMSC) [26,28], adipose-derived stem cells (ADSC) [20,26,28], and stem cells from human exfoliated deciduous teeth (SHED) [33,34] have also been tested for dental pulp regeneration. 

There have been increasing attempts to regenerate the pulp–dentin complex by transplanting mesenchymal stem cells in animal studies [19,20,21,22,23,24,25,26,27,28,29,30,31,33,34] and clinical trials [32,34]. The aim of this study was to systematically review the performance of various types of mesenchymal stem cells for dental pulp regeneration and their tissue engineering protocols for clinical translation and applicability.

## 2. Results

### 2.1. Study Selection Process

**Figure 1:**
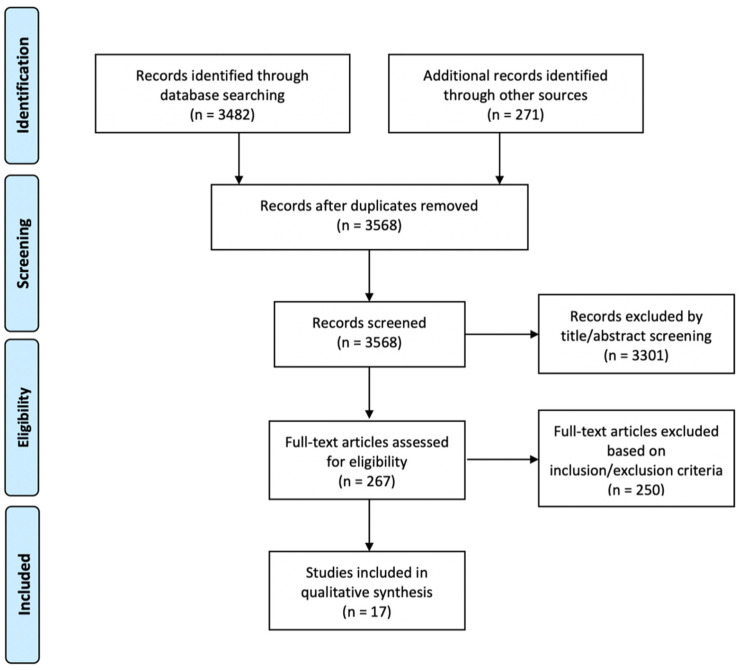
The Preferred Reporting Items for Systematic Reviews and Meta-Analyses (PRISMA) flow chart.

Electronic and hand searching generated 3568 articles, of which 267 were included for full-text review for relevance after title and abstract searching. A total of 17 studies met the inclusion criteria. Fifteen animal studies [19,20,21,22,23,24,25,26,27,28,29,30,31,33,34] and two clinical studies [32,34] were included for this review. The selection processes are described in Figure 1. 

### 2.2. Study Characteristics of the Included Animal Studies

The characteristics of the 15 animal studies are summarized in Table 1. 

#### 2.2.1. Animal Models 

All included studies except for one [27] used large animal models for orthotopic de novo pulp regeneration [19,20,21,22,23,24,25,26,28,29,30,31,33,34]. A dog model (73%) [19,20,21,22,23,24,25,26,28,29,30] was found to be the most common, followed by a pig model (20%) [31,33,34]. A rat model was used in one study [27]. 

#### 2.2.2. Tooth Types

Incisors were the most preferred tooth type [19,20,21,22,23,24,25,26,28,31,33], followed by premolars [29,30,31] for large animal models. Molars were used in a small animal model [27]. Twelve studies used incisors [19,20,21,22,23,24,25,26,28,31,33], while three studies used premolars [29,30,31]. One study used both incisors and premolars in a large animal model [31]. 

#### 2.2.3. Presence of the Previous Infection

All studies except for one study [19] included no previous root canal infection before cell transplantation. In one dog study [19], root canal infection and apical periodontitis were induced by mechanical disruption of pulp tissue and supragingival plaque placement. The root canals were disinfected with sodium hypochlorite and antibiotic paste (metronidazole, ciprofloxacin, and minocycline) before the transplantation of autologous DPSC [19]. All other studies [20,21,22,23,24,25,26,27,28,29,30,31,33,34] performed pulpectomy before cells were transplanted, without induction of root canal infection.

#### 2.2.4. Types of Transplanted Mesenchymal Stem Cells

Autologous cells were used most commonly for cell transplantation [19,20,21,22,23,26,28,29,30,31,34]. Allogeneic cells were used in two dog studies [24,25] and one pig study [31], while autologous cells were used in 10 dog studies [19,20,21,22,23,26,28,29,30,34] and two pig studies [31,34]. In two studies [27,33], human stem cells were also transplanted into the root canals of rat molars or pig incisors. One study used both allogeneic and autologous cells in a pig model [31]. 

Among the 15 studies included, 13 studies used DPSC (five studies) [19,27,29,30,31] and its subpopulations (eight studies) [20,21,22,23,24,25,26,28]. Of the eight studies using DPSC subpopulations [20,21,22,23,24,25,26,28], mobilized DPSC (MDPSC) isolated by granulocyte colony stimulating factor (G-CSF) induction were used most commonly (six studies) [21,22,23,24,25,28]. Pulp CD105+ cells and CD31- SP cells were other DPSC subpopulations used in two studies [20,26]. Deciduous pulp stem cells (SHED) were also used in two pig studies [33,34]. The subpopulations of BMSC were used in two studies [26,28]. Mobilized BMSC (MBMSC) [28] isolated by granulocyte colony stimulating factor (G-CSF) induction and bone marrow CD31- SP cells [26] were used as BMSC subpopulations in dog studies. ADSC subpopulations were also used in three studies for pulp regeneration [20,26,28]. Adipose CD105+ cells [20] and CD31- SP cells [26], as well as mobilized ADSC (MADSC) [28] isolated by granulocyte colony stimulating factor (G-CSF) induction, were used as ADSC subpopulations in dog studies. 

#### 2.2.5. Biomaterial Scaffolds

All animal studies except for two [33,34] used biomaterial scaffolds [19,20,21,22,23,24,25,26,27,28,29,30,31]. The most common scaffolds were collagen-derived materials such as atelocollagen [21,22,23,24,25,28], or collagen TE (collagen tissue equivalent; a mixture of collagen type I and type III) [20,26,31], used in nine studies [20,21,22,23,24,25,26,28,31]. Chitosan hydrogel [19], hyaluronic acid gel [31], platelet-rich plasma (PRP) [29,30], and nanofibrous spongy microsphere–poly(L-lactic acid) [27] were used as scaffolds for cell transplantation. Two pig studies [33,34], without introducing biomaterial scaffolds into root canals, used the aggregates of deciduous pulp stem cells containing extracellular matrix, which could serve as a scaffold. 

#### 2.2.6. Signaling Molecules

Nine studies used signaling molecules [19,20,21,22,23,24,25,26,28], of which G-CSF was most commonly delivered with cells (six studies) [21,22,23,24,25,28]. Stromal derived factor-1 was used in two studies [20,26], and a cocktail of growth factors including vascular endothelial growth factor-2 (VEGF-2), basic fibroblast growth factor (bFGF), platelet-derived growth factor (PDGF), nerve growth factor (NGF), and bone morphogenetic protein-7 (BMP7) was used in one study [17]. No signaling molecules were introduced into the root canals in six studies [27,29,30,31,33,34].

#### 2.2.7. Time after Transplantation

Histological assessment was performed in samples over varying time periods, ranging from two weeks to 9 months after transplantation. The most frequent time point for the histological analysis was approximately three months (nine studies) [20,21,23,24,29,30,31,33,34], followed by two weeks (six studies) [20,21,22,25,26,28]. 

#### 2.2.8. Histological Findings

Tissues formed in the root canals after cell transplantation include vascularized pulp-like tissue (13 studies) [19,20,21,22,23,24,25,26,27,28,31,33,34], dentin-like tissue on the native dentin (10 studies) [19,20,21,22,23,24,25,26,28,31], odontoblast-like cells (11 studies) [20,21,22,24,25,26,27,28,31,33,34], and nerve fibers (nine studies) [20,21,22,24,25,26,28,33,34]. Vital tissues resembling periodontal tissue such as cementum-like, periodontal ligament-like, and bone-like tissue were observed in a study using PRP as a scaffold, regardless of cell transplantation [29,30]. Allogeneic transplantation of DPSC or MDPSC showed regeneration of pulp-like and dentin-like tissues similar to those of autologous transplantation [20,21,23,24,25,28,31].

The differences in regeneration potential were dependent on the specific subsets of stem cells delivered with signaling molecules. When SDF-1 was used as a signaling molecule, pulp CD105+ cells showed greater pulp tissue regeneration than adipose CD105+ cells or total pulp cells [20], while pulp CD31- SP cells also showed higher amounts of regenerated pulp tissue than bone marrow CD31- SP cells [26]. When G-CSF was used, MDPSC showed higher degrees of regeneration, vascularization, and innervation than MBMSC and MADSC [28]. MDPSC transplantation with G-CSF showed less inflammation and apoptosis and a higher degrees of tissues and nerve regeneration than groups without G-CSF [21].

The regeneration of blood vessels was clearly presented in all fifteen studies. Immunostaining with protein gene product (PGP) 9.5 antibody showed nerve fibers in the regenerated tissues in dog studies [20,21,22,24,25,26,28]. Nerve regeneration was identified with the overlapping neuronal marker and nuclear staining after SHED transplantation in a pig study [34]. Furthermore, regenerated blood vessels and nerves were not only from host endogenous cells but also from transplanted pulp cells based on the positive staining with antihuman nuclear and mitochondria antibodies in another pig study [33].

The reduction in regeneration potential was observed in aged dogs. There was approximately 60% regenerated area in root canals of aged dogs (5 to 6 years old) after 120 days [22], whereas there was more than 90% in young dogs (8 to 10 months old) after 60 days when MDPSC was transplanted [21]. The entire pulp tissue was found to be regenerated at 90 days after MDPSC transplantation [23]. The trypsin pretreatment of pulpectomized root canals prior to MDPSC transplantation resulted in three times higher regeneration of pulp-like tissue in aged dogs than no trypsin pretreatment [25]. 

Increased regeneration associated with hypoxia was reported in a rat study [27]. DPSC precultured under hypoxic conditions before transplantation regenerated more blood vessels than DPSC precultured under normoxic conditions [27]. No or minimal tissue regeneration was observed in groups without cell transplantation in two dog studies [19,23].

### 2.3. Study Characteristics of the Included Clinical Studies

The characteristics of the two clinical studies are summarized in Table 2. 

#### 2.3.1. Study Design 

One pilot clinical trial [32] and one randomized controlled trial [34] were conducted to regenerate pulp using autologous stem cells. In a pilot clinical trial [32], five patients with irreversible pulpitis with ages ranging from 20 to 44 years old were recruited for autologous MDPSC transplantation into root canals. An untreated normal tooth was used as a control in each patient. MDPSC were isolated from the patients’ third molars by G-CSF induced mobilization and expanded in vitro in a culture medium supplemented with autologous serum in a Good Manufacturing Practice (GMP)-compliant facility. The autologous MDPSC suspended in an atelocollagen scaffold and G-CSF were transplanted into the disinfected root canals. After tooth restoration, patients were followed-up for clinical and radiographic evaluations.

In a randomized controlled trial [34], 40 patients with traumatized necrotic incisors with ages ranging from 7 to 12 years old were recruited and randomly assigned to either the autologous pulp stem cell transplantation group (30 patients, experimental group) or the apexification group (10 patients, control group). For the autologous human deciduous pulp stem cell (hDPSC) isolation, maxillary deciduous canines were extracted. The hDPSC were isolated and expanded in GMP-grade reagents. The expanded hDPSC aggregates were transplanted into disinfected root canals for patients in the experimental group and apexification was performed for patients in the control group. Three patients in the experimental group were excluded due to loss of follow-up, while one patient was excluded due to tooth fracture. A total of 36 patients were followed-up for clinical or radiographic assessments.

#### 2.3.2. Clinical and Radiographic Findings

In the pilot clinical trial [32], no adverse events were reported in five patients who received autologous MDPSC transplantation for 24 weeks. There were no postoperative symptoms during follow-ups. No periapical radiolucency was detected in three cases at follow-up. One premolar case with a periapical lesion developed after enrollment showed a decrease in the size of the lesion after MDPSC transplantation during follow-ups. Another premolar case showed an increase in the size of periapical radiolucency over the follow-up period. All patients showed positive responses to electric pulp testing. Cone-beam computed tomography (CBCT) analysis showed dentin formation in three teeth. Magnetic resonance imaging (MRI) revealed complete regeneration of pulp-like tissue in all cases based on the finding that the signal intensity was similar to that of normal pulp. 

In the randomized controlled trial [34], 36 patients received full analysis using radiovisiography (RVG), CBCT, laser Doppler flowmetry, and electric pulp testing. CBCT analysis showed a significantly higher increase in root length, apical foramen width, and dentin thickness in the hDPSC transplantation group (experimental group) than in the control group. No periapical inflammation was observed by RVG during follow-ups. The experimental group showed functional responses to electric pulp testing and vascular formation evidenced by laser Doppler flowmetry. No adverse events were observed in 20 patients for 24 months after hDPSC transplantation. 

### 2.4. Appraisal of Study Quality

**Figure 2:**
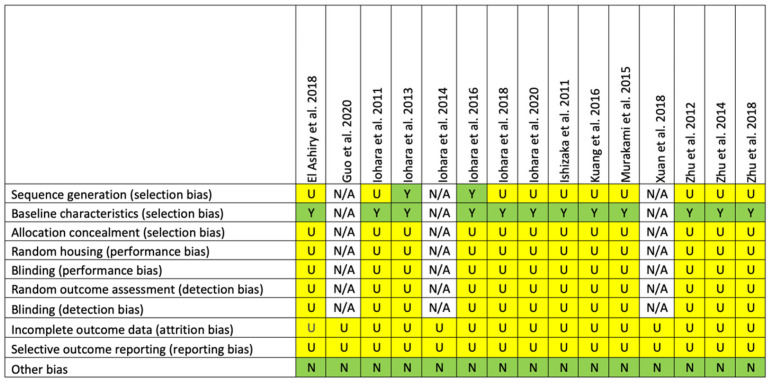
Risk of bias assessment for the included animal studies. N/A indicates that there was only one group. U: unclear; Y: yes; N: no.

**Figure 3:**
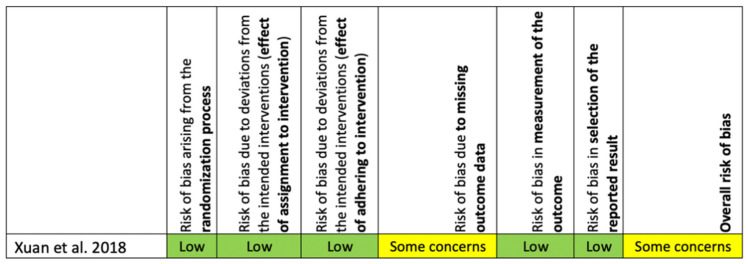
Risk of bias assessment for the randomized controlled trial using the Cochrane Risk of Bias (RoB 2.0) tool.

**Figure 4:**
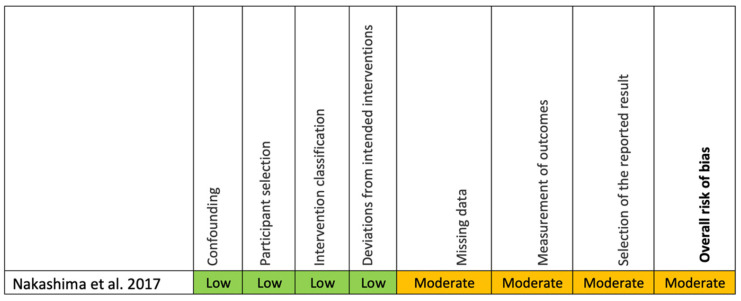
Risk of bias assessment for the clinical trial using the Risk of Bias in Non-Randomized Studies of Interventions (ROBINS-I) tool.

Figure 2, Figure 3 and Figure 4 depict the risk of bias assessment for 15 animal studies and two clinical trials. The Systematic review center for laboratory animal experimentation (SYRCLE) risk of bias tool was used to assess the included animal studies (Figure 2). Regarding selection bias, only two animal studies [21,23] used random allocation, while all studies except for three that involved only one group had similar characteristics among groups at baseline. None of the included studies described whether the allocation of the animals to groups was blinded. Assessment of performance bias revealed that none of the studies reported whether animals were randomly housed within the animal rooms and whether investigators were blinded during the experiment. Regarding detection bias, none of the studies mentioned whether animals were selected randomly for outcome assessment and whether outcome assessors were blinded. The risk of attrition and reporting bias was unclear in all studies. There were no other sources of bias that affected the results in the studies. The overall risk of bias in animal studies was unclear due to the lack of reporting of experimental details. 

The Cochrane Risk of Bias tool [35] was used to assess a randomized controlled trial and ROBINS-I tool (38) was used to assess a pilot clinical trial. The overall risk of bias of the randomized controlled trial showed that there was one domain (risk of bias due to missing outcome data) judged to have some concerns, without a high risk of bias for any other domains (Figure 3). For the pilot clinical trial, the overall risk of bias was moderate because the risk of bias in all domains was low or moderate (Figure 4). 

## 3. Discussion

Dental pulp regeneration has been a clinically approved therapy by the American Dental Association since January 2011 [4]. However, there is still an unmet clinical need for regeneration of pulp tissue, which is particularly important for patients with various degrees of pulpal and periapical infection. There have been a multitude of studies showing that pulp regeneration therapy with evoked bleeding and no stem cell transplantation has yielded tissues mimicking periodontal tissue, such as cementum, bone, and periodontal ligament [36,37,38]. The goal of pulp regeneration therapy for diseased teeth is to reconstitute the pulp–dentin complex [3,4], and the current clinical protocols may fail to achieve this goal [5]. 

There have been several systematic reviews concerning stem-cell-based pulp regeneration [39,40,41]. The previous systematic reviews identified pulp and dentin regeneration in their included studies using in situ stem cell transplantation strategies [39,40,41], as in this study. The present scoping review included several more current studies based on eligibility criteria than the previous systematic reviews. With the data analysis involving more studies, this review could provide more up-to-date information focusing on tissue engineering protocols for the clinical translation and applicability of a cell-based approach to dental pulp regeneration. A meta-analysis was not performed in the present review due to the significant heterogeneity of animal models, transplanted cells, scaffolds, and growth factors among the included studies. 

Successful tissue regeneration is driven by the tissue engineering triad, including stem cells, biomaterial scaffolds, and signaling molecules [6]. The usage of mesenchymal stem cell transplantation for pulp regeneration has recently attracted increasing attention due to promising reported outcomes. This scoping review has focused on orthotopic de novo pulp regeneration in animal studies and clinical trials because ectopic animal models using cell transplantation in renal capsules [42] or subcutaneous tissues [43,44] cannot simulate local microenvironments that significantly contribute to the regenerative processes of transplanted stem cells, and thus are less clinically relevant. A total of 17 studies that used de novo orthotopic pulp regeneration models, including 15 animal studies and two clinical trials, were identified through the selection processes. All animal studies using stem cell transplantation except for two studies that used a PRP scaffold [29,30] revealed the regeneration of vascularized pulp-like tissue. Autologous cell transplantation in two clinical studies [32,34] also successfully regenerated vascularized pulp-like tissue, as revealed by the findings from pulp vitality testing, CBCT, or MRI. 

The presence of the previous infection may affect stem cell functions and regeneration outcomes because it significantly alters regenerative processes by devastating essential regenerative microenvironments and constructive stem or progenitor cells [45]. Indeed, the histologic observations from human and animal studies have shown that tissues formed in the previously infected canals are mostly of periodontal origin, such as cementum, bone, and periodontal ligament, rather than of pulpal origin [36,37,38,46,47]. In the present review, however, one animal study using an infection model with the induction of apical periodontitis showed robust pulp and dentin regeneration when autologous DPSC were transplanted [19]. More animal studies with an infection model should be conducted to prove whether the same efficacy of pulp regeneration identified in the present review could be achieved in the infection model.

Large animal models have been preferred in biomedical research due to better suitability for clinical translation [48,49]. Compared with a rodent model, dogs and pigs are considered more translatable models based on their biochemical, genetic, and physiological similarities to humans [31,48,49,50,51]. A dog model was identified to be the most common, followed by a pig model for orthotopic pulp regeneration in this review. Only one study used a rat model for cell transplantation in molars [27]. Incisors and premolars are most widely used in all large animal studies because these tooth types have more anatomical resemblance to human teeth and are more accessible when performing endodontic therapy than more posteriorly located molars. 

The use of autologous cells is ideal for stem cell transplantation therapy because it circumvents safety issues, such as possible immune rejection and pathogen transmission between donors and recipients [52]. As shown in this review, autologous stem cells have been used in most animal studies and all clinical trials. However, autologous cell transplantation still encounters major hurdles for clinical translation, such as donor site morbidity, difficulties with cell isolation and ex vivo expansion, loss of cells and stemness during cryopreservation or banking, need for a GMP facility, and regulatory and economic barriers [4,53]. Allogeneic stem cell transplantation is proposed as an alternative strategy, and the efficacy of allogeneic stem cells for orthotopic pulp regeneration has been reported in several animal studies [24,25,31]. However, allogeneic stem cell transplantation suffers from immune-related problems as well as difficulties and barriers related to autologous cell transplantation [4,53]. No clinical trials with allogeneic stem cells for pulp regeneration have been identified yet. 

**Figure 5:**
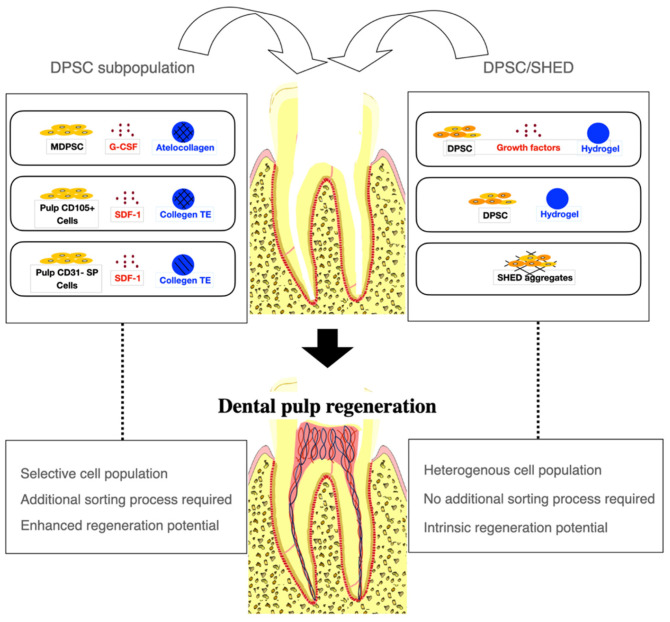
A cell-based approach to dental pulp regeneration. Dental pulp stem cell (DPSC) subpopulations have been most commonly used for dental pulp regeneration to augment regenerative processes. However, an additional cell sorting process is required. DPSC and Stem cells from human exfoliated deciduous teeth (SHED) have been used with or without growth factors or scaffold materials. Robust pulp and dentin regeneration has been observed in animal studies using either DPSC subpopulations or DPSC, although the efficacy of regeneration may be enhanced with DPSC subpopulations. MDPSC: mobilized dental pulp stem cells; G-CSF: granulocyte-colony stimulating factor; SDF-1: stromal-derived factor-1; Collagen TE: collagen tissue equivalent (mixture of collagen type I and type III).

The selection of appropriate stem cells for cell transplantation is critical for successful pulp regeneration (Figure 5). The majority of the studies included in this review used DPSC subpopulations to augment the regenerative processes. Of the eight studies using DPSC subpopulations [20,21,22,23,24,25,26,28], MDPSC were used most commonly [21,22,23,24,25,28,32]. In five dog studies by Iohara et al. [21,22,23,24,25], MDPSC were isolated from autologous DPSC by G-CSF-induced chemotaxis for cell transplantation. The transplantation of MDPSC is thought to be advantageous for pulp regeneration because they release trophic factors that promote angiogenesis and neurogenesis and have high immunosuppressive properties [21,22,23,24,25]. Histological analysis showed that pulp-like tissue with vasculatures and nerves and dentin-like tissue with odontoblast-like cells were regenerated when MDPSC were transplanted [21,22,24,25]. The efficacy of regeneration was found to be significantly higher when MDPSC were transplanted with G-CSF compared with MDPSC alone or G-CSF alone [21]. Attenuation in inflammation and apoptosis was also observed when MDPSC and G-CSF were delivered together [21]. Another dog study by Murakami et al. [28] transplanting MDPSC also showed similar robust pulp–dentin regeneration with vasculature and nerves. In a clinical trial by Nakashima et al. [32], MRI revealed complete pulp regeneration in teeth with irreversible pulpitis when MDPSC were transplanted with G-CSF. The safety and feasibility of the use of autologous MDPSC and G-CSF for pulp regeneration were demonstrated in a pilot clinical trial [32]. Pulp CD105+ cells and CD31- SP cells were also shown to release a higher amount of angiogenic and neurotrophic factors and regenerated significantly higher volumes of vascularized and innervated pulp-like tissue than stem cells from other tissue origins [20,26]. In a dog study by Iohara et al. [20], greater pulp tissue regeneration was observed when pulp CD105+ cells were delivered compared with adipose CD105+ cells. In another dog study by Ishizaka et al. [26], pulp CD31- SP cell transplantation yielded significantly higher pulp regeneration than bone marrow CD31-SP cell transplantation. The transplantation of autologous, deciduous pulp stem cells for pulp regeneration has been shown to be safe and effective in an animal study and a randomized controlled trial. Other mesenchymal stem cells, such as subpopulations of BMSC and ADSC, have been suggested as alternative stem cell sources for pulp regeneration [20,26,28].

The incorporation of biomaterial scaffolds can facilitate the attachment, proliferation, and differentiation of transplanted stem cells. Injectable scaffolds such as hydrogels are highly desirable for successful pulp regeneration therapy because of their excellent conformity to the complex root anatomy and readiness of stem cell and growth factor delivery, while providing a structural framework for cellular functions [54]. Most of the included studies used collagen-derived scaffolds, among which atelocollagen was the most common type [21,22,23,24,25,28,32]. Atelocollagen is a collagen solution that can be easily introduced into root canals with stem cells and growth factors, forming a hydrogel under physiological conditions [21]. A clinical-grade atelocollagen scaffold was safely used in a pilot clinical trial [32]. Chitosan, which can be manufactured in the form of hydrogels, has been used for various regenerative applications in skin, cartilage, bone, fat, and nerve due to its favorable physical properties, such as its porosity for cellular growth and nutrient transport and its fluid absorption capacity, biodegradability, biocompatibility, non-immunogenicity, and antimicrobial activity [55]. Lyophilized chitosan hydrogels used in animal studies for pulp regeneration [19,56] employed the benefits of chitosan’s physicochemical properties in tissue regeneration, while allowing vascularization within the scaffold material [55]. PRP has been used for pulp regeneration therapy [29,30,57,58]. It can be useful for tissue regeneration since it contains multiple growth factors, such as PDGF, fibroblast growth factor, transforming growth factor β, and vascular endothelial growth factor [57]. However, the use of PRP as a scaffold did not aid in the regeneration of the pulp–dentin complex, as evidenced by the ectopic tissue formation in animal studies [29,30,59]. Therefore, caution should be exercised with the use of a PRP scaffold for pulp regeneration. 

The use of signaling molecules is beneficial for regenerative therapy, since it can enhance the cellular activities of transplanted stem cells toward regeneration. In the present review, G-CSF was found to be most commonly used for pulp regeneration [20,21,22,23,24,25,32]. G-CSF has been originally approved by the United States Food and Drug Administration for use to decrease infection in patients with immunosuppressive cancer therapy [60]. For dental pulp regeneration, the use of G-CSF was proposed because regenerative biological events such as angiogenesis, neurogenesis, cell migration and proliferation, antiapoptosis, and immunosuppression were augmented by G-CSF [61,62]. The efficacy of the use of G-CSF coupled with MDPSC for pulp regeneration was shown in a pilot clinical trial [32] and several animal studies [20,21,22,23,24,25]. One animal study used a cocktail of growth factors including VEGF-2, bFGF, PDGF, NGF, and BMP7 for pulp regeneration by orchestrating cellular behaviors of transplanted DPSC and showed successful regeneration of vascularized pulp-like and dentin-like tissue [19]. Six studies did not use any exogenous signaling molecule because it was presumed that trophic factors released by transplanted stem cells, as well as local biological cues from conditioned dentin and resident cells, could serve as endogenous signaling molecules [27,29,30,31,33,34]. 

Complete pulp regeneration after stem cell transplantation may take approximately three months in animal models [23], although it appears to heavily depend on the age of the animals [22]. Trypsin pretreatment or use of nanobubbles may rescue age-dependent decline in regeneration potential [25]. Hypoxia priming of transplanted stem cells may be beneficial in promoting pulp regeneration by increasing vasculogenesis and odontoblastic differentiation [27]. 

This scoping review demonstrated that the regeneration of pulp-like tissue, dentin-like tissue, and odontoblast-like cells lining the dentin, blood vessels, and nerves could be achieved using the stem cell transplantation strategy in animal models. Furthermore, similar regeneration may be achieved in patients, as shown in clinical trials using autologous stem cells. From a clinical perspective, the tissue engineering protocols used in de novo orthotopic pulp regeneration models in the present review may be useful for translation into therapeutic applications. The analysis of data from the two clinical trials may also provide clinicians with more information to better implement a cell-based approach to dental pulp regeneration and bring this strategy to the forefront of patient care. However, the findings from this scoping review should be interpreted in the context of the following limitations. First, the overall risk of bias observed in animal studies was unclear and the overall risk of bias in clinical trials was moderate or judged to raise some concerns. Second, the long-term safety and efficacy of stem cell transplantation have not been reported yet. The longest follow-up period in the included studies was 24 months [34] for clinical trials and nine months [20] for animal studies. High-quality animal studies are needed to facilitate the clinical translation of this stem cell transplantation approach. More well-designed randomized controlled trials are also required to determine the safety and efficacy of the therapeutic applications of mesenchymal stem cells for dental pulp regeneration. 

## 4. Materials and Methods

### 4.1. Search Strategy

This review was conducted and reported according to the Preferred Reporting Items for Systematic Reviews and Meta-Analyses (PRISMA) statement [63]. Electronic searches for relevant studies were performed in the PubMed, Scopus, Web of Science, Embase, and Medline databases from their inception until January 2021 using the combinations of the following keywords: “pulp regeneration”, “pulp revascularization”, “pulp revitalization”, “regenerative endodontics’, “animal”, “human”, “clinical”, “stem cells”, “mesenchymal stem cells”, “pulpectomy”, “cell transplantation”. In addition, hand searches were conducted from the reference lists of the identified review articles. 

### 4.2. Eligibility Criteria and Study Selection

The articles were screened based on the following inclusion or exclusion criteria. Inclusion criteria were as follows: animal studies, clinical trials, pulpectomy performed, mesenchymal cell transplantation into the root canal space, and orthotopic transplantation models. Exclusion criteria were as follows: in vitro studies, review papers, case reports, mesenchymal cells not transplanted into the root canal space, and ectopic transplantation models. Studies were identified initially by title and abstract and finally by full text for inclusion in the review.

### 4.3. Data Extraction

The following data were extracted from the included studies: animal models, tooth types, types of transplanted mesenchymal cells, biomaterial scaffolds, signaling molecules, and histological findings for animal studies, and study types, types of transplanted cells, tooth types, biomaterial scaffolds, signaling molecules, follow-up time, and clinical and radiographic findings.

### 4.4. Appraisal of Study Quality 

The risk of bias for the individual studies was assessed using the Systematic Review Center for Laboratory Animal Experimentation (SYRCLE) guidelines [64] for animal studies, the Cochrane Risk of Bias (RoB 2.0) tool [35], and the Risk of Bias in Non-Randomized Studies of Interventions (ROBINS-I) tool [65] for clinical studies.

## 5. Conclusions

Stem cell transplantation is a major in vivo pulp regeneration approach. DPSC and their subpopulations have served as the main mesenchymal stem cell sources for pulp regeneration due to their high trophic and immunomodulatory effects, as well as their tissue specificity. The use of autologous stem cells has been suggested to be more clinically translatable, as it avoids possible immune-related issues. The safety and efficacy of autologous and allogeneic stem cell transplantation have been shown in animal studies. In two clinical trials, autologous pulp stem cell transplantation generated vascularized and innervated pulp-like tissues, as shown by pulp vitality testing and imaging analyses, and their safe application was confirmed with follow-ups for up to two years. There still remain barriers, such as difficulty with cell isolation and ex vivo expansion, the need for a cell banking system and a GMP facility, and regulatory approval, which are yet to be overcome for clinical translation. More high-quality randomized clinical trials are needed to further confirm the safety and efficacy of the stem cell transplantation strategy for dental pulp regeneration. 

## Figures and Tables

**Table 1:** Summary of the characteristics of the included animal studies.

Study	Animal Models	Tooth Types	Presence of Previous Infection	Types Transplanted Stem Cells	Biomaterial Scaffolds	Signaling Molecules	Time after Transplantation	Main Histological Findings
El Ashiry et al., 2018 [19]	Dog	36 incisors from 12 dogs	Yes	Autologous DPSC	Chitosan hydrogel	VEGF-2, bFGF, PDGF, NGF, BMP7	4 months	Vascularized pulp-like tissue and dentin-like tissue. No tissue regeneration was observed in growth factor + scaffold only group.
Guo et al., 2020 [33]	Pig	Incisors	No	Human SHED aggregates	N/A	N/A	3 months	Vascularized pulp-like tissue, odontoblast-like cells, and nerve fibers. The regenerated blood vessels and nerves were found to be partially originated from transplanted human SHED based on the positive immunocytochemical staining with antihuman nuclear and mitochondria antibodies.
Iohara et al., 2011 [20]	Dog	60 incisors from 15 dogs	No	Autologous pulp CD105^+^ cells or adipose CD105^+^ cells	Collagen TE (mixture of collagen type I and type III)	SDF-1	14 days, 28 days, 90 days	Vascularized pulp-like tissue, dentin-like tissue with odontoblast-like cells, and nerve fibers. Pulp CD105^+^ cells + SDF-1 group showed significantly higher regenerated tissue than adipose CD105^+^ cells + SDF-1 group or total pulp cells + SDF-1 group.
Iohara et al., 2013 [21]	Dog	72 incisors from 18 dogs	No	Autologous DPSC (DPSC subpopulation isolated by G-CSF induced mobilization)	Atelocollagen	G-CSF	14 days, 60 days, 90 days	Vascularized pulp-like tissue, dentin-like tissue with odontoblast-like cells, and nerve fibers DPSC + G-CSF group showed significantly higher volume of regenerated tissue than DPSC only group or G-CSF only group. Attenuation of inflammatory cells and apoptotic cells and increased nerve regeneration were observed in groups with G-CSF than in groups without G-CSF.
Iohara et al., 2014 [22]	Dog	16 incisors from 4 dogs	No	Autologous MDPSC (DPSC subpopulation isolated by G-CSF induced mobilization)	Atelocollagen	G-CSF	14 days, 120 days	Vascularized pulp-like tissue, dentin-like tissue with odontoblast-like cells, and nerve fibers. Tissue regeneration potential was age-dependent. Aged dogs showed ~60% regeneration volume after 120 days. Note that young dogs showed more than 90% after 60 days (Iohara et al. 2013).
Iohara et al., 2016 [23]	Dog	28 incisors from 5 dogs	No	Autologous MDPSC	Atelocollagen	G-CSF	1 day (MRI only), 90 days, 180 days	Vascularized pulp-like tissue and dentin-like tissue. The entire root canal was filled with regenerated tissue at 90 days after cell transplantation. Collagen scaffold only group showed minimal tissue.
Iohara et al., 2018 [24]	Dog	19 incisors from 14 dogs	No	Allogeneic MDPSC	Atelocollagen	G-CSF	12 weeks	Vascularized pulp-like tissue, dentin-like tissue with odontoblast-like cells, and nerve fibers. No significant difference was observed in regenerated tissues between MDPSC transplantation groups with the matched and mismatched dog leukocyte antigen. Dual transplantation showed similar tissue regeneration to single transplantation.
Iohara et al., 2020 [25]	Dog	25 incisors from 12 dogs	No	Allogeneic MDPSC	Atelocollagen	G-CSF	14 days, 36 weeks	Vascularized pulp-like tissue, dentin-like tissue with odontoblast-like cells, and nerve fibers. Higher volume of pulp-like tissue was observed in aged dogs after trypsin pretreatment. The toxicology assessment showed the safety of trypsin pretreatment. The use of nanobubbles with trypsin pretreatment enhanced vascularization.
Ishizaka et al., 2011 [26]	Dog	30 incisors from 10 dogs	No	Autologous pulp, bone marrow, adipose CD31^-^ side population (SP) cells	Collagen TE	SDF-1	14 days, 28 days	Vascularized pulp-like tissue, dentin-like tissue with odontoblast-like cells, and nerve fibers. Pulp CD31^-^ SP cells + SDF-1 group showed significantly higher regenerated tissue than bone marrow CD31^-^ SP cells + SDF-1 group. There was no significant difference in regenerated pulp area between pulp CD31^-^ SP cells and adipose CD31^-^ SP cells.
Kuang et al., 2016 [27]	Rat	12 molars from 6 rats	No	Human DPSC	Nanofibrous spongy microsphere/poly(L-lactic acid)	N/A	4 weeks	Vascularized pulp-like tissue and odontoblast-like cells. Hypoxia-primed DPSC group showed more vascularity and odontoblast-like cell formation than normoxia group.
Murakami et al., 2015 [28]	Dog	20 incisors from 5 dogs	No	Autologous MDPSC or MBMSC or MADSC (subpopulations isolated by G-CSF induced mobilization)	Atelocollagen	G-CSF	14 days	Vascularized pulp-like tissue, dentin-like tissue with odontoblast-like cells, and nerve fibers. MDPSC group showed significantly higher amounts of regeneration, vascularization, and innervation than MBMSC and MADSC group.
Xuan et al., 2018 [34]	Pig	Incisors from 3 pigs	No	Autologous deciduous pulp stem cell (SHED) aggregate	N/A	N/A	3 months	Vascularized pulp-like tissue, odontoblast-like cells, and nerve fibers NeuN-positive cells were found in regenerated tissue after cell transplantation, but were not found in normal pulp tissue.
Zhu et al., 2012 [29]	Dog	16 premolars from 4 dogs	No	Autologous DPSC	Platelet-rich plasma (PRP)	N/A	3 months	Vital tissue (soft connective tissue), cementum-like tissue, periodontal ligament-like tissue, and bone-like tissue in most of cases. No significant difference was found between groups with or without DPSC transplantation and between groups with or without PRP.
Zhu et al., 2014 [30]	Dog	16 premolars from 4 dogs	No	Autologous DPSC	Platelet-rich plasma (PRP)	N/A	3 months	Vital tissue (soft connective tissue), cementum-like tissue, periodontal ligament-like tissue, and bone-like tissue (the same study as Zhu et al., 2012, with additional histological analyses). Immunohistochemical and histochemical analyses showed no difference among blood clot group, PRP group, DPSC group, and DPSC + PRP group.
Zhu et al., 2018 [31]	Pig	Incisors, premolars from 5 pigs	No	Allogeneic or autologous DPSC	Hyaluronic acid gel or collagen TE gel	N/A	1.5–4 months	Vascularized pulp-like tissue, dentin bridge, osteodentin, and dentin-like tissue with odontoblast-like cells. Allogeneic DPSC regenerated tissue similar to those of autologous DPSC.

DPSC: dental pulp stem cells; SHED: stem cells from human exfoliated deciduous teeth; MDPSC: mobilized dental pulp stem cells; MBMSC: mobilized bone marrow stem cells; MADSC: mobilized adipose-derived stem cells; VEGF-2: vascular endothelial growth factor-2; bFGF: basic fibroblast growth factor; PDGF: platelet-derived growth factor; NGF: nerve growth factor; BMP7: bone morphogenetic protein-7; SDF-1: stromal-derived factor-1; G-CSF: granulocyte-colony stimulating factor; MRI: magnetic resonance imaging.

**Table 2:** Summary of the characteristics of the included clinical studies.

Study	Study Types	Types Transplanted Cells	Tooth Types	Biomaterial Scaffolds	Signaling Molecules	Follow-Up Time	Main Clinical and Radiographic Findings
Nakashima et al., 2017 [32]	Pilot clinical trial	Autologous MDPSC (DPSC subpopulation isolated by G-CSF-induced mobilization)	5 teeth with irreversible pulpitis (3 premolars, 2 incisors)	Atelocollagen	G-CSF	1, 2, 4, 12, 24, 28, 32 weeks	No adverse events were observed for 24 weeks. There were no clinical symptoms up to 24 weeks. No periapical radiolucency was found in three cases. One case with a periapical lesion prior to transplantation had a periapical radiolucency, which gradually decreased in size during follow-ups. Another case showed an increase in periapical radiolucency during the follow-ups. Positive electric pulp testing was observed in all patients during follow-ups. Complete pulp regeneration in the apical and coronal part of the root canal was observed based on the signal intensity of MRI, similar to that of normal, untreated pulp. Functional dentin formation was found in three teeth using CBCT.
Xuan et al., 2018 [34]	Randomized controlled trial	Autologous deciduous pulp stem cell (hDPSC) aggregate	Traumatized incisors with pulp necrosis;26 teeth: hDPSC;10 teeth: apexification	N/A	N/A	1, 2, 3, 6, 9, 12, 24 months	The hDPSC group showed significantly higher increases in root length, apical foramen width, and dentin thickness than the apexification group based on the CBCT analysis. An increase in vascular formation was identified in the hDPSC group by laser Doppler flowmetry. Electric pulp testing showed a significantly higher decrease in sensation in hDPSC group than apexification group. No adverse events were observed for 24 months.

MDPSC: mobilized dental pulp stem cells; DPSC: dental pulp stem cells; hDPSC: human deciduous pulp stem cells; G-CSF: granulocyte-colony stimulating factor; CBCT: cone-beam computed tomography.

## Data Availability

Not applicable.
